# The Sanatorium Treatment of Consumption

**Published:** 1904-03-05

**Authors:** T. N. Kelynack

**Affiliations:** Physician to the Mount Vernon Hospital for Consumption and Diseases of the Chest, Hampstead and Northwood, London.


### Hospital Clinics and Medical Progress.

### THE SANATORIUM TREATMENT OF CONSUMPTION.

By T. N. Kelynack, M.D., M.R.C.P, Physician to the Mount Vernon Hospital for Consumption and Diseases of the Chest, Hampstead and Northwood, London.

(*Continued from Page* 389.)

### The Development of British Sanatoria.

Although to Bodington may be given the distinction of having been the first to establish a sanatorium in England, on what we now term open-air lines, it cannot be denied that the main impetus which of recent years has done so much to revolutionise treatment, has come to us from Germany. Brehmer established Gorbersdorf, in Silesia, in 1854, but the teaching of Walther and the methods employed at Nordrach, have, I think, had most directing influence in moulding action in relation to hygienic treatment in this land.

The time, however, has now come when a slavish imitation of German procedure is likely to hamper rather than hasten progress in this country.

Not a few superintendents of sanatoria in Great Britain and Ireland have been patients at Nordrach, or have been greatly influenced by the masterly personality of Walther, and no doubt sentiment as well, perhaps, as a willingness to comply with popular tendencies has led to the not altogether unobjectionable practice of naming certain British sanatoria after the name of the far-famed Black Forest resort.

It is remarkable also how many English sanatoria, in their usually elaborately-illustrated prospectuses, insist on the fact that the "Nordrach system" is faithfully followed.

A slavish imitation of any so called system is almost necessarily stultifying to the scientific habit of mind, and the rational therapeutist can have but little sympathy with the claims of those who seek popularity by pandering to the whims of those who favour a fashionable resort to foreign procedures without carefully considering if they are based on sound principles, conducted on rational lines, and suited to the particular needs of the given situation.

Without discussing the point at length, I think it will be generally admitted that there is much, or at all events, has been much in the German methods of dealing with consumptives which is not only unscientific, but unnecessary, and peculiarly unsuited to the character, constitution and custom of most British patients.

The fact is that, as our general hospitals and other institutions have found, what succeeds abroad does not necessarily prove best at home. And in the institutional treatment of consumption this has been recognised by our best observers. If progress is to be maintained we should remember that much in our present methods of procedure is in great measure experimental, and we must be prepared to revise, modify, amplify, or even discard as experience and experiment may indicate.

There are remarkable contrasts in the sanatoria of this country. Some are substantial places, elaborate in design, luxurious in fitting, expensive in conduct, and altogether prohibitory except to the wealthy. Others are composed of flimsy huts, ephemeral chalets, and transient makeshifts of shelters which, like the Bedouin's tent, may readily pass away into the region of things that cease to be.

In many cases our sanatoria are situated in elevated, protected, dry, sunny and picturesque districts, while in not a few instances they of necessity have been established in comparatively low, unprotected, damp, dull and uninteresting country.

But as far as one can gather, where strict hygienic conditions are enforced and thorough medical supervision provided, the results do not show any such variation as might be considered as essentially dependent on merely local conditions. After visiting sanatoria in all parts of the British Isles, some situated in particularly sheltered nooks and others located in wild and wind-swept positions, not a few furnished with almost all the luxuries of club residence, while many necessitate a vigorous exposure requiring Spartan fortitude, I find that, speaking generally, provided there is a strict adherence to hygienic methods combined with constant and systematic medical supervision, the results appear to be almost equally satisfactory.

### The Educational Aspect of Sanatorium Life.

Although some Reports of British Sanatoria still persist in giving returns of so-called "cures"—and it may be noted that the returns of cases in which apparent arrest of the disease has been attained vary from 16·4 per cent. in public institutions to 85 per cent. in privately-conducted establishments [15] —it is manifest that in many cases, and particularly in those where on the grounds of expense or other reasons the patients are unable to remain in a sanatorium for more than a few months, restoration can only be imperfect. This being so the educational aspect of sanatorium life deserves the most thoughtful consideration. Many a case by a comparatively short residence in a properly-conducted institution is started on the road to permanent arrest of the disease, and is taught not only the details of practice necessary for recovery, but the main duties of the hygienic life on the fulfilment of which future safety depends.

In this connection I may be allowed to make reference to the interesting educational work which this summer I was able to observe being carried out under the direction of Dr. R. W. Philip, at Craigleith, the sanatorium for the consumptive poor of Scotland, where Edinburgh cases are allowed to attend as "day-boarders," and receive instruction as to the best management of their "night-quarters" at home, from a visiting medical officer.

The remarkable municipal experiment now being conducted at Brighton under the supervision of Dr. Arthur Newsholme is one also which has interested me much and which, after personal investigation, I venture to affirm, no sanitarian can afford to neglect.

Among the sanatoria for comparatively poor cases which I have recently inspected, I found one where

[15] See tables given by Dr. Arthur Latham in appendices to his Prize Essay, 1903.

the visiting physician, a lady medical, was in the habit of giving a weekly lecture to her patients on the conduct of a healthy life and with such results, I was assured, that many became enthusiastic missionaries in the cause of hygienic righteousness.

Not only in many sanatoria but in dispensaries for consumptive cases, and out-patient departments, through visiting by district nurses and health visitors, and in many other ways short and sensible instruction is being given which, although certainly not systematic and perhaps not altogether scientific, is nevertheless accomplishing much in the education of public opinion.

And in this matter since open-air methods have attracted popular fancy and in great measure become fashionable, we must not forget the important part which the press has taken in awakening both conscience and intelligence to the needs of the situation.

It is sometimes urged that visitors should not be encouraged to come to a sanatorium. On almost every ground I am opposed to the strict isolation of consumptives in sanatoria far removed from their homes, as some would advise. I believe, and for physiological and psychological as well as humanitarian reasons, that a weekly visiting day is beneficial for an institution, and I am sure an establishment readily accessible to large centres of population and open, under of course proper restrictions, to the friends of the patients and supporters of the institution, furnishes one of the best means of breaking down that unfortunate view which, perhaps in consequence of a not sufficiently guarded enthusiasm in the conduct of prophylactic measures, has tended to make the consumptive an outcast from society. A sanatorium should stand as a constant object lesson of the necessity of living a hygienic life, and may well act as a warning to those who would neglect the prophylactic and curative virtues of natural methods.

### The Principles of Sanatorium Treatment.

We are wont to speak glibly of the principles whereby we are directed in our sanatorium treatment, but after a careful study of much of recent literature dealing with these so-called principles and an extensive investigation of the actual details of hygienic management as carried out in various institutions in Great Britain and Ireland, I am compelled to conclude that most of these principles are so ill-defined or ill-understood as to afford but scant direction for any action which might be considered as determined by scientific precision.

As a matter of fact, much if not most in sanatoria treatment is at the present time mainly empirical. In almost all our methods of procedure we are still in the experimental stage. Most discordant views prevail, as I have already indicated, regarding the pathology of pulmonary tuberculosis, and widely divergent opinions are promulgated respecting its origin, propagation, prevention, and arrest.

We know that the so-called natural factors in sanatorium treatment, if rightly applied, are instrumental in securing benefit for the patient, but we do well to honestly admit that we are still far from understanding much of the why and the wherefore. Considerable research will be necessary before we can claim to enunciate fully the principles on which successful treatment must depend. It is well that we do not deceive ourselves by a misuse of terms. Our ignorance should stimulate us to inquiry. It is much to be regretted that but few of the sanatoria in this country publish any thoroughly scientific reports or are undertaking anything worthy of being designated systematic, scientific, original research. And yet innumerable questions call for answer and many problems await solution. Much work remains to be done concerning the conditions of the blood in phthisical cases,[16] varying states of nutrition, influence of exercise, fatigue and rest, dietetic considerations, and many other points which will occur to everyone of you.

I find that there are remarkable differences in the employment of clinical methods. Perhaps the most important is that which concerns the registration of temperature.[17] In some institutions the old axillary method is still employed. Many consider records taken in the rectum essential for guidance, while the majority use the oral procedure, but apparently often neglect the precautions necessary for a reliable reading. At some sanatoria great importance is given to "exercise" temperatures, while others, and as I think wisely, place chief reliance on registration during rest. Some few sanatoria superintendents consider $x$-rays useful for diagnostic purposes. In one sanatorium I found the sputum was always weighed. The formation of weekly average charts is recommended by some.

### Dietetic Considerations.

In visiting various sanatoria one is amazed and amused at the divergent opinions and practice regarding the all-important matter of feeding. Wide differences exist, theoretical and practical; but it must be admitted good results seem to follow in spite of discordance. In one sanatorium all the cases are stuffed with meat both cooked and raw. In another it is sought to conduct all patients back to health on a vegetarian diet. Serious attempts are being made in several institutions to place the arrangements of diet on a scientific basis.[18]

It is satisfactory to find that the irrational method of forced feeding now holds but little sway in this country, but in discarding such practice it is necessary to remember that the phthisical subject seems to require what is practically hyper-alimentation as compared with the healthy man under like conditions of rest and exercise. Some sanatoria have discarded the routine administration of cod-liver oil, and use instead considerable quantities of milk, cream and butter, and apparently with much advantage.

### Industrial Colonies for Consumptives.

Sanatoria treatment, as now generally conducted in this country, may accomplish much, but it is becoming manifest that in many ways modification and extension is desirable. The practical physician has need to study the psychology of the consump-

[16] See Halbron on "Le Sang dans la Tuberculose," *Revue de la Tuberculose*, October, 1903; and H. G. Pesel's article in *Medical Press and Circular*, Oct. 28th, 1903.

[17] *British Medical Journal*, October, 1903.

[18] See articles by J. J. Galbrath in *British Medical Journal*, March 14th, 1903; N. D. Bardswell and J. E. Chapman in *British Medical Journal*, March 7th, 1903. J. Mitulescu furnishes an exhaustive paper on food stuffs in tuberculosis in *Zeit. f. Tub. u. Heilstätten*, Bd. IV. Hf. 6, August, 1903. Consult "Die Chemische Pathologie der Tuberculose." Edited by Dr. A. Ott. Berlin: 1903.

tive. This matter has recently very rightly received considerable attention.[19]

The manner of life pursued by patients in many sanatoria is wont to engender a selfishness of disposition and habit of idleness which in only too many cases renders the patient little better than a troublesome parasite. The moral deterioration which an improperly conducted institutional life is so apt to develop is giving, as I well know, much cause for anxiety to many conscientious superintendents of sanatoria in this country. I am convinced that much in the course of life initiated and maintained as a part of sanatoria treatment proves detrimental to altruistic tendencies and leads to the development of selfishness and actual sins born of idleness. On medical as well as moral and economic grounds the movement to provide suitable employment for cases undergoing treatment deserves encouragement.

During a recent visit to the interesting Kelling Sanatorium, at Holt, in Norfolk, I found many of the early cases were encouraged to engage in gardening, and even in some cases were allowed to use wheelbarrows, and I was assured that no ill results had occurred. It is certainly most necessary that in connection with our sanatoria for the poor in this country adequate provision should be made for the employment of the working consumptive in suitably constructed and conducted workshops, and every effort should be made to encourage an out-of-doors occupation as is done at the Beelitz Sanatorium, near Berlin.[20] There is much need for the founding of industrial colonies for consumptive subjects in this country.[21] In at least one sanatorium where children are taken, arrangements are made whereby open-air schooling may be carried on. If only medical practitioners would themselves undertake the rôle of patient for say a week I venture to think much useful extension and modification in some sanatoria would result.[22]

[19] See *Archives de Neurologie*, Paris, January, 1903; and article by G. A. De Santos Saxe in *New York Medical Journal*, August 1st, 8th, 1903.

[20] *British Medical Journal*, April 4th, 1903.

[21] "Suggestions on Industrial Colonies for Consumption," by Claude C. Chidell, M.B., *Tuberculosis*, October, 1902.

[22] The *Practitioner*, September, 1903.

(*To be concluded.*)

